# A Review of Methods for Increasing the Durability of Hot Forging Tools

**DOI:** 10.3390/ma18153669

**Published:** 2025-08-04

**Authors:** Jan Turek, Jacek Cieślik

**Affiliations:** 1 Faculty of Economics, Finance and Transport, Podkarpacka Szkoła Wyższa, ul. Na Kotlinę 8, 38-200 Jasło, Poland; jturek@psw.jaslo.pl; 2 Faculty of Mechanical Engineering and Robotics, AGH University of Krakow, Al. Mickiewicza 30, 30-059 Kraków, Poland

**Keywords:** hot-work forging tools, alloy structural steels, welded dies, zone surfacing, surfacing materials, wear mechanisms, tool regeneration, forging die design, computer modeling of forging processes, forging process efficiency

## Abstract

The article presents a comprehensive review of key issues and challenges related to enhancing the durability of hot forging tools. It discusses modern strategies aimed at increasing tool life, including modifications to tool materials, heat treatment, surface engineering, tool and die design, die geometry, tribological conditions, and lubrication. The review is based on extensive literature data, including recent publications and the authors’ own research, which has been implemented under industrial conditions at the modern forging facility in Forge Plant “Glinik” (Poland). The study introduces original design and technological solutions, such as an innovative concept for manufacturing forging dies from alloy structural steels with welded impressions, replacing traditional hot-work tool steel dies. It also proposes a zonal hardfacing approach, which involves applying welds with different chemical compositions to specific surface zones of the die impressions, selected according to the dominant wear mechanisms in each zone. General guidelines for selecting hardfacing material compositions are also provided. Additionally, the article presents technological processes for die production and regeneration. The importance and application of computer simulations of forging processes are emphasized, particularly in predicting wear mechanisms and intensity, as well as in optimizing tool and forging geometry.

## 1. Introduction

One of the main challenges for the industry in the modern world is to concentrate efforts on producing high-quality products at minimal costs [1]. A significant and challenging issue in die forging is the low durability of forging tools [2,3,4,5,6,7,8,9,10,11,12,13,14,15,16,17,18,19,20,21,22,23,24,25,26,27,28,29,30,31]. During forging, processes occur that cause geometric changes to the surface and structure, as well as residual stresses in the surface layer of the die material [23]. The high intensity of wear on plastic-forming tools makes the problem of tool durability a significant issue, and increasing attention is being paid to it [12,13,14,15,16,17,18,19,20]. The phenomena responsible for die wear are complex and depend on factors related to the die itself, the working conditions of the dies, the material, and the shape of the forgings [12,13,14,15,16,21,22,23,24,25,26,27]. As the tool wear process progresses, mainly expressed (in the case of friction wear) by changes in its dimensions and shape, the quality of the product deteriorates; products with greater dimensional deviations, and worse surface quality, and even products with clear defects in the form of scratches and tears caused mainly by build-ups on the tools are obtained [26]. The low durability of forging tools is primarily caused by the extreme conditions present in hot forging processes [26]. These conditions involve the simultaneous occurrence of many complex phenomena and destructive mechanisms. Such mechanisms result from external forces and environmental influences acting on the die surfaces [20,25]. The phenomena responsible for die wear are highly complex and depend on multiple factors. The most important are related to die design, which is determined by the shape and dimensions of the produced forgings, as well as by the operating conditions. From scientific, technological, and economic perspectives, durability remains a challenging problem to solve [23,32]. The wear of forging equipment during use generates significant costs. These expenses constitute a considerable portion of the overall production costs. Currently, tool costs are estimated to account for as much as 14–16% of total production costs and, in extreme cases, in the case of small production series, even 31%. In reality, taking into account the time needed to replace worn-out tooling or in the case of unexpected tool destruction, these costs can increase to as much as 42%. In addition, tool wear significantly reduces the quality of manufactured forgings. Currently, there are no clear criteria for the evaluation and selection of methods to improve the durability of the tools [23,33,34]. Only general directions are known; they should be adapted to the implementing processes. The shape of the tool cutting edge also influences the intensity of the occurrence of individual destructive mechanisms, strength properties (also at elevated temperatures), microstructural features, and properties of the surface layer of the tool material [23]. The surface layer of forging dies should be characterized by an appropriate stereometric structure and especially by low roughness and a low coefficient of friction (only in some applications is a high coefficient of friction beneficial), high resistance to abrasive and fatigue wear, and good resistance to oxidation and corrosion [23]. Currently, several methods and technologies are employed to enhance the durability of forging tools. The competitive market environment forces forging manufacturers to continuously reduce costs and produce high-quality forgings, which results in significant interest in the problem of short tool life [2,3,4,5,6,7,8,9,10,12,13,14,15,16,17,18,23,33,34,35,36,37,38]. Therefore, scientific and research and development centers use a number of IT tools and advanced research and also develop modern technologies that allow both analysis and increase the “durability” of forging tools [33]. The study of destructive mechanisms and other phenomena occurring and forecasting the durability of tools in forging processes is a complex issue, and many works have been written [2,10,11,20,23,25,33,35,36,39]. Similar works related to improving the durability of dies can be found in numerous scientific publications [5,10,11,12,13,14,15,16,22,23,26,27,28,29,30,31,32,33,34,35,40]. The basic mechanism of destruction of the working surfaces of die inserts in forging processes is abrasive wear—approximately 70%. The other mechanisms are deformation plastic and thermo-mechanical fatigue [34]. The subject of abrasive wear mechanisms was dealt with by many authors, including [41], while the problem of the influence of thermal load cycles on tool wear was included in [26,42]. Automation of the forging process and an important contribution to understanding the wear mechanisms taking place were presented in [43].

## 2. Methods for Improving the Durability of Forging Dies

The durability of forging dies depends mainly on the deformations occurring in the forging process, the temperature, and the rate of deformations (which determine the nature of thermo-mechanical loads) but also on the type of tool material and heat and surface treatment [12,18,23,25,35,44]. Various analyses and scientific studies have been conducted on the influence of individual destructive mechanisms on the wear of forging tools in forging processes in which some research centers take the position [25,33] that abrasive wear dominates [45], while others believe that the greatest degradation is caused by cracking due to thermomechanical fatigue [46].

**Figure 1:**
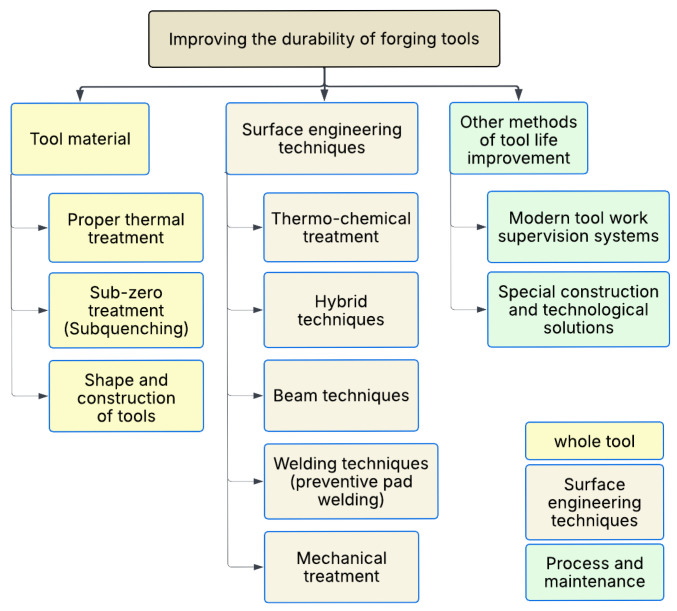
Tool life improvement methods. Reproduced with permission from Ref. [33].

Currently, economic considerations, especially the high labor intensity of making forging dies and the significant amount of tool materials used, dictate the need to solve several design and technological problems correctly. An important issue is the high price of hot-work tool steels due to the significant share of alloying elements. Modifying the surface layer properties using various engineering methods of forging tools is the most effective way to improve their durability [23,26,47]. Many examples are confirming that the durability of forging tools can be effectively increased by hardfacing [18,23,38,47]. The authors have developed a new design of the matrix technology and a change of material properties, using their own, original concept of replacing the basic materials from which die cubes are manufactured—using traditional hot-work tool steels—with 42CrMo4 alloy structural steel. This material, when appropriately heat-treated, has sufficient strength to transfer thermo-mechanical loads occurring during forging of a wide range of forgings, especially on presses. It is obvious that in order to obtain high durability, it is necessary to appropriately modify the properties of the surface layer of the working surfaces of the dies: for this purpose, the surface of the cuts was refined by surfacing using wires made of materials with an appropriately selected chemical composition [23]. New surfacing materials with different and better functional properties have great potential for use in many types of dies used on presses and even hammers. Examples of such steels with improved functional properties include 55H3SMF—a Cr-Mo-V tool steel with added sulfur for enhanced machinability and impact resistance—and 41HMFS—a modified version of the 42CrMo4 alloy with vanadium and sulfur to improve hardenability, wear resistance, and machinability. Another direction of improving durability is to attempt to develop and use new groups of materials in the production of hot-work forging tools, e.g., steels with high resistance to tempering or hardened martensitic steels. A threat to the durability of dies is the overheating of tools during the forging processes [48]. Therefore, when observing the technological process, it is necessary to control the temperature and cool the tools [44]. Forging tools are made of hot-work tool steel. The surface temperature of the tools in contact with the metal is usually between 200 and 400 °C and can locally reach up to 600 °C. An alternative material is ceramic forging dies [49], which improve the durability of tools. Ceramics can also be used to strengthen the surface of tools [36]. Ceramic inserts can be mounted in holders placed inside the tools in places of the most intensive wear, which strengthen places exposed to too strong effects of high temperature [4,39]. Tribological conditions [38] are particularly important because excessive friction causes abrasive wear of tools [8]. Studies have been carried out on the appropriate preparation and surface topography of tools to ensure the appropriate tribological conditions [50]. Ensuring the best tribological conditions affects the quality of die durability [38]. There are other methods of improving die durability and quality that have emerged recently [1]. Six Sigma is one of the most practical and effective techniques for improving tool durability. A commonly used technique for implementing Six Sigma is the DMAIC approach (Define, Measure, Analyze, Improve, and Control) [1], which is used to increase productivity and quality and also to make the hot forging process robust to quality differences. Many researchers have applied Six-Sigma-based techniques to study and improve a wide range of industrial processes [2]. Cutting costs and reducing the time consumed become a difficult task. Hence, it is necessary to study and optimize the process parameters of hot forging operations [51]. Methods of increasing durability related to the modification of tool material, heat treatment, surface layer, tooling design, tool shape, and tribological conditions are presented in [25]. The durability of forging tools can be improved using various methods (Figure 1, after [33]).

These methods can be classified as follows:

1. Methods related to the entire tool (selection of tool material and appropriate heat treatment and optimization of tooling shape and design);
2. Methods related to the surface layer (hybrid techniques, thermochemical treatment, and welding and mechanical methods);
3. Other methods not directly related to the tool (supervision systems allowing for full monitoring of the process and solutions allowing for effective extension of the service life of forging tools).

Various methods can be employed to enhance the operational durability of forging tools:

1. Design measures include but are not limited to the following:
  – Selection of the chemical composition of the tool material, enabling the production process to obtain the appropriate microstructure and properties throughout the volume and within the surface layer of the manufactured tool;
  – Proper design of forgings;
  – Proper selection of the number and type of die cuts and the correct design of their stereometric shape and dimensions as well as surface roughness characteristics.
2. Technological means, which include the use of advanced methods of shaping, finishing, and surface treatment, such as plastic working, abrasive and electrochemical treatment (electro-polishing), burnishing (to impart high smoothness and introduce ultimate compressive stresses into the surface layer), heat and thermochemical treatment, and electro-spark strengthening.
3. Operating measures, which include, among others, the use of running-in and appropriate lubrication and cooling media, as well as ensuring the proper technical condition of forging machines and devices, compliance with the dates of periodic inspections, and other recommendations of the manufacturers of equipment and materials used in the production process.

In particular, the following is advisable:

– To use high-alloy, wear-resistant, and thermo-mechanical fatigue-resistant tool steels;
– To design multi-element forging dies assembled with working elements made of materials with high mechanical properties;
– To use strengthening surface treatment, e.g., burnishing.

**Figure 2:**
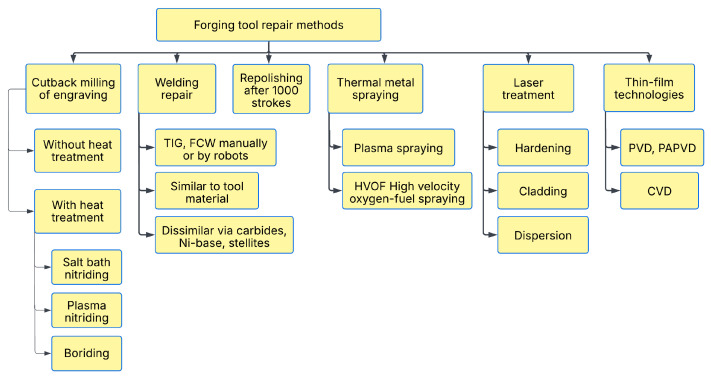
Established repair methods used for forging. After from Ref. [5].

Important factors influencing the durability of forging tools are die material, die design, die manufacturing parameters, and forging conditions, which can influence the appropriate type of failure or damage. As presented in [52,53], there are practical repair methods to extend the useful life of the tool or reduce tooling costs. Depending on the actual wear mechanism, hard coatings, including nitriding, are an appropriate means to increase abrasion resistance, while stellites perform better under high thermal load. The application of forging tool repair technology (improvement of durability) in practice is shown in Figure 2 (after [5]).

### 2.1. Durability of Forging Tools in Relation to the Chemical Composition of Hot-Work Tool Steels

**Figure 3:**
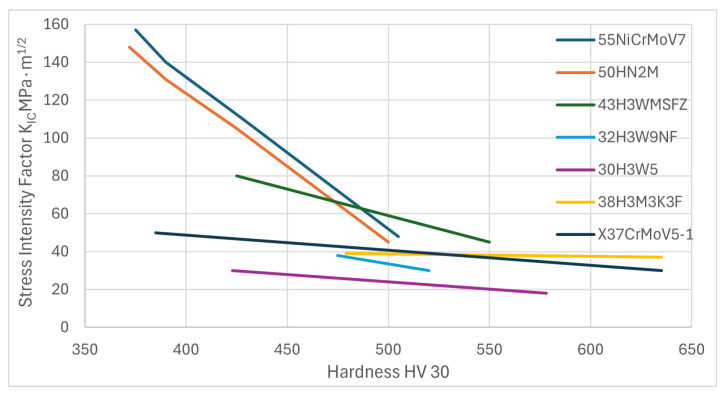
The effect of hardness on the resistance of some steels, evaluated using the Stress Intensity Factor K_IC_, MPa·m^1/2^. Reproduced with permission from Ref. [58].

The issue of selecting the optimum chemical composition of the steel used in hot work is complex due to the diverse working conditions of the tools made of these steels and the associated various optimization criteria. These criteria are established after analyzing the dominant wear mechanisms of individual tool groups and the resulting requirements for the utility of steel. For example, the chemical composition of the steel intended for the production of tools, for which the dominant wear is abrasive wear at elevated temperatures, should be different from the composition of steels used for the production of forging tools, which are primarily required to be highly resistant to dynamic loading. It is difficult and often impossible to formulate an unambiguous criterion for the durability of a specific group; therefore, a method is used, which consists of optimizing the conditions ensuring the maximum value of the property determining the durability of tools, assuming that other properties also affecting the durability cannot be lower than certain values considered to be minimal. The optimization procedure uses experiment planning methods and multistep regression analysis to determine approximate mathematical relationships between factors that influence the selected steel property [54]. Despite certain simplifications, the method presented here allows, with a correctly selected optimization criterion, the development of a chemical composition of steel with utility properties better than those currently used. As a result of numerous laboratory tests and operational trials, it was determined that the optimum carbon concentration in steel for various groups of hot-work tools ranges from 0.3% C to 0.5% C. Increasing the concentration of this element to 0.6% is used in low-alloy steels in which carbon is the main component that determines the increase in hardenability, strength, and abrasion resistance [6,55] and some multi-component steels [56]. As a result of the optimization of the chemical composition of steels intended for the production of large forging dies, it was found that more favorable properties than the commonly used 55NiCrMo4 type steel, for example, 50H2N2MF type [57], 50H2MNF type [38], 50H2NMFS type, and 44HN3MF type [20], have more favorable properties than the previously commonly used 55NiCrMo4-type steel. These steels, in comparison with 55HNN2M-type steel, usually contain less carbon but more carbide-forming elements, which is why they exhibit higher strength properties at elevated temperatures while maintaining high impact strength. We used 50H2N2MF-type steel in the forging plant (for MPM-16000 hammers) instead of 55NiCrMo4 steel (WNL) and observed a 2–3% increase in the durability of the forging dies. Steels with nickel have good impact strength at room temperature, as our research has shown, but at elevated temperature, this property is comparable to the impact strength of other nickel-free steels [55]. With a proper explanation of tools and proper heating before starting work, the use of nickel-containing steel is often unjustified. Especially when the required hardness exceeds 480HV, nickel-free high-alloy steels have better fracture resistance (Figure 3, after [58]).

Therefore, nickel-free steels, including large-sized chromium–manganese steels with additions of molybdenum, vanadium, silicon, and boron, are increasingly used to manufacture forging tools. Steels of the 55H3SMF type, 50H2 SF type [41], and 41HMFS type [6,20] have been developed. The forging dies used in presses are made of high-alloy steels. These steels should be characterized by high resistance to tempering, thermal fatigue, and abrasion at elevated temperatures; therefore, compared to low-alloy steels, they must contain a significantly higher concentration of carbide-forming elements: chromium, molybdenum, tungsten, and vanadium [20]. The optimal carbon concentration ranges from 0.3% C to 0.4% C [38]. This results from a sufficient crack resistance and thermal fatigue resistance, and these properties deteriorate with increasing carbon concentration in steel [59]. The concentration of alloying elements is difficult to optimize because the effect on functional properties depends on many factors. Based on the research conducted so far, it can be stated that the optimal concentration of chromium ranges from 2.5% to 5.0% Cr, molybdenum ranges from 1.0% to 5.0% Mo, and vanadium ranges from 0.5% to 1.5% V [6,20]. Tungsten, which is often a component of hot-work tool steel, significantly increases its resistance to tempering and thus its strength properties at elevated temperatures. However, tungsten reduces thermal conductivity, plastic properties, and thermal fatigue [59] and should, therefore, not be used as a basic alloying addition in hot-work steels. The required properties of hot-work steels can be obtained with different concentrations of basic alloying elements, depending on the assumed optimization criteria. The most commonly adopted criterion is the maximum resistance to thermal fatigue at elevated temperatures while maintaining resistance to tempering, whereas the criterion of resistance to corrosive effects is less frequently adopted. In the selection of the tool material, manufacturers such as Uddeholm-Böhler (Voestalpine), Schmolz, Bickenbach, and others supply a number of different types of steel [25]. Among them are steels with an increased content of alloying elements, which increases their hardness while maintaining similar ductility. The manufacturers of such steels are Dievar, Hotvar, Unimax, and Termodur. Research is also being conducted on the development of new types of steel for hot work. These studies concern the chemical composition of steel [52], alternative methods of obtaining it by casting instead of forging ingots [32], and semi-permanent forming [54]. Research focuses on the properties of steel before wear [32] and after the forging process [42]. A good example of an effective application is the method used by the authors to change the tool material in the technological process of forging from X37CrMoV5-1 steel to Unimax. Under the conditions of one-time forging, they obtained an increase in durability of 150% [15].

### 2.2. The Influence of Design and Technology on the Production of Dies and Forging Equipment and Errors in the Technological Process

The design work includes proper selection of the number and type of die cuts and correct design of their stereometric shape and dimensions, as well as surface roughness characteristics [47]. Proper design of dies and selection of the chemical composition of the tool material allow for the manufacturing process of the appropriate microstructure and properties across the entire volume and within the surface layer of the manufactured tool design to be achieved [38]. In the literature, many tips can be found on designing forging tools using numerical modeling [40,49]. In the work [9], principles and guidelines for the design of forging tools are included. Another aspect influencing the durability of tools in forging processes is the construction of auxiliary equipment [32]. It ensures the rigidity of the entire set of tools. Hot-work forging tools are made by erosion treatment (cut cutting shape) or (most often) by machining, and then they are subjected to heat treatment and finishing (abrasive) and sometimes thermochemical treatment (e.g., nitriding) or other technological operation of the surface layer [38]. Technological activities related to the tool include the use of advanced shaping, finishing, and surface processing methods, such as plastic processing, abrasive and electrochemical processing (electro-polishing), burnishing (to obtain high smoothness and introduce ultimate compressive stresses into the surface layer), heat and thermochemical treatment, and electro-spark hardening [47]. In the production process, design errors can be found related to the selection of incorrect radii in elements subjected to heat treatment [38]. There are also technological errors related to the lack of coherence with the machine package. Damage caused by the incorrect shape of transitions (sharp edges instead of radii) after heat treatment shows hardening cracks [33]. The problem that occurs in the process of manufacturing dies and forging equipment is damage that causes premature wear during operation [38]. This damage occurs (of the working surface of the dies) during improper transport from individual stations or mechanical processing.

### 2.3. Influence of the Heat Treatment Process and Defects Occurring

**Figure 4:**
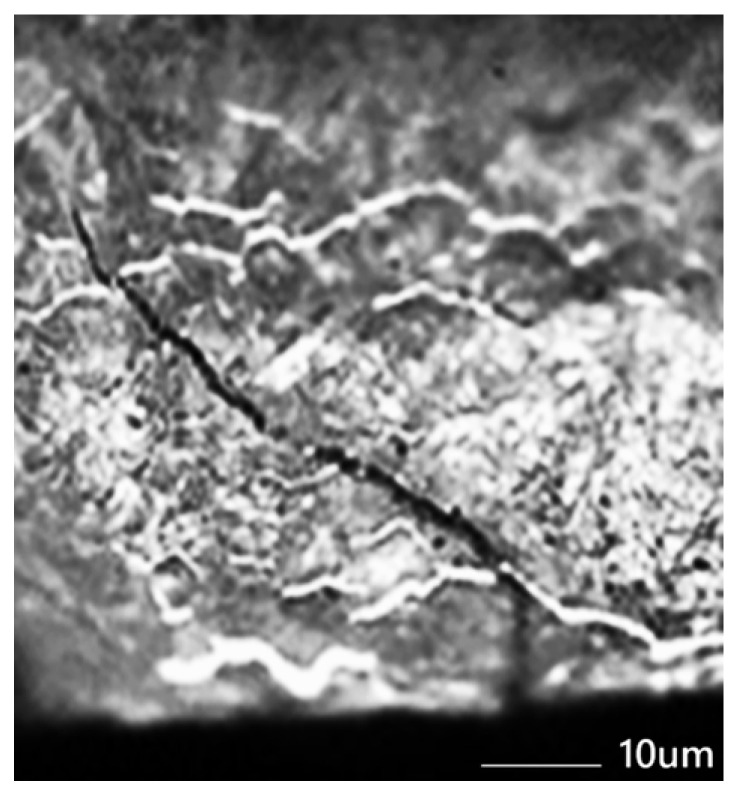
Improper thermal treatment—a crack along grain boundaries revealed on tool surface after nitriding [34].

Heat treatment is one of the key factors that influences the durability of forging dies. The use of even the most expensive types of tool steel for hot work, without a properly selected and carefully performed heat treatment, usually does not provide satisfactory results [38]. Many valuable studies on this subject are available in the technical literature. First of all, technological recommendations for heat treatment, developed by manufacturers of specific steel types, among others, should be strictly observed. Failure to follow them can lead to premature tool damage—in the form of cracks in the case of excessive hardness or permanent plastic deformations in the case of too soft a structure. The key property of tool steels, which is abrasion resistance, is largely determined by the presence of a large number of fine, evenly distributed carbides (both cementite and alloy carbides) in a properly hardened forging die [47]. Equally important is ductility, understood as the tool’s resistance to dynamic loads, which is particularly important in hot-work conditions. This property is largely dependent on the correct selection of the heat treatment parameters [60]. The articles [8,61] present the possibilities of heat treatment. Treatment at temperatures below zero (cold or cryogenic treatment) deserves attention, which also applies to steel reinforced with hot work [40,48]. The purpose of such treatment (technological process) is to remove the retained austenite from the steel microstructure. The mentioned treatment improves the strength of the material and increases the dimensional stability of the tools produced in this way [62]. Heat treatment defects are mainly the result of failure to meet the complex parameters of their production heat treatment [38]. In order to improve steel through changes in structure caused by phase transformations occurring in the solid state, heat treatment operations are used in tools for hot-plastic working. Heat treatment operations consist of three procedures: annealing (depending on), hardening, and single, double, or triple tempering [63]. The tempering temperature should be higher than the working temperature of the tools [47]. The authors confirm that industrial practice has confirmed double and most often triple tempering, e.g., in steel: X37CrMoV5-1 (WCL) or X40CrMoV-5-1-1 (WCLV). An example of incorrect heat treatment is a crack that runs along the grain boundaries, revealed on the surface of a tool subjected to gas nitriding (Figure 4).

The following main disadvantages of the heat treatment of steels for hot-work tools can be mentioned, which determine the durability of tools [16,25]:

1. Decarburization and oxidation of the surface during prolonged heating at high temperature.
2. Excessive austenite grain growth and the associated reduction of plastic properties and impact strength caused by too high a temperature (overheating) and long heating time during austenitizing.
3. Loss of the secondary hardness effect after tempering and reduced tempering resistance. These defects arise due to low saturation of austenite with alloying elements and carbon during hardening from too low a temperature (underheating).
4. Low-strength properties caused by the formation of microstructures consisting of carbides and ferrite not saturated enough with alloying elements. This defect is caused by too slow cooling from the austenitizing temperature.

### 2.4. Engineering Methods for Improving the Surface Layers of Forging Dies

The surface layer requirements for forging tools, particularly dies, generally differ from those imposed on other process components [47,64]. Due to the dynamic development of research in the field of die durability and the emergence of new methods replacing outdated solutions, it has become necessary to systematize the available knowledge on durability enhancement techniques. The literature distinguishes several key categories of methods aimed at improving tool longevity, including modification of tool materials, appropriate heat treatment, optimization of tool shape and design, tribological conditions, and surface treatment or surface engineering techniques [3,52]. The surface layer of the forging dies should exhibit a proper stereometric structure, most notably, low surface roughness and low coefficient of friction (although in specific applications, a higher coefficient may be beneficial), as well as high resistance to abrasive and fatigue wear, and good resistance to oxidation and corrosion [38]. Among surface finishing methods, electropolishing and chemical polishing are particularly noteworthy [64]. However, surface engineering techniques are considered the most promising for improving wear resistance as they directly reinforce the areas most exposed to degradation [24,38]. In recent years, surface engineering has developed rapidly, providing numerous advanced solutions in the form of protective layers and coatings [16]. Consequently, these methods play a crucial role in extending the operational life of forging tools. An innovative method in the field of surface treatment is the use of hybrid technologies, which provide a broad spectrum of process types that allow the purchase of a wide range of materials with unique applications for many specific applications that cannot be obtained using standard surface treatment methods [44,65]. Currently, various methods and technologies are available to enhance the surface layer and increase the durability of forging tools. Modifying the properties of the surface layer of the forging tools is the most effective way to improve their durability [66]. Modifying the properties of the surface layer of the tools is possible thanks to the development of surface engineering methods, e.g., surfacing [38]. The article [67] presents directions for the development and implementation of hybrid technologies in surface engineering. In general, techniques for shaping the properties of the surface layer can be divided into the following categories [65]:

– Techniques involving thermochemical treatment (diffusion layers);
– CVD and PVD techniques;
– Mechanical techniques (e.g., burnishing, shot peening, and discing);
– Beam techniques (e.g., ion implantation and laser processing);
– Hybrid techniques.

**Figure 5:**
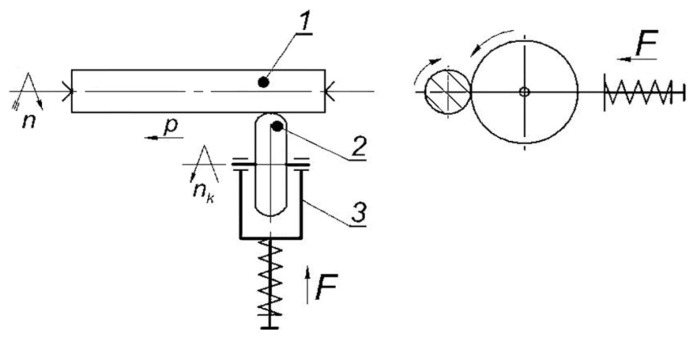
Schematic diagram of the rolling burnishing of the punch using a device with a spring clamp. The burnishing rolling tool is placed in the support (the moving part of the lathe that moves the tool along the workpiece): 1—machined punch, 2—disc, and 3—body of the device mounted in the lathe holder [23].

**Figure 6:**
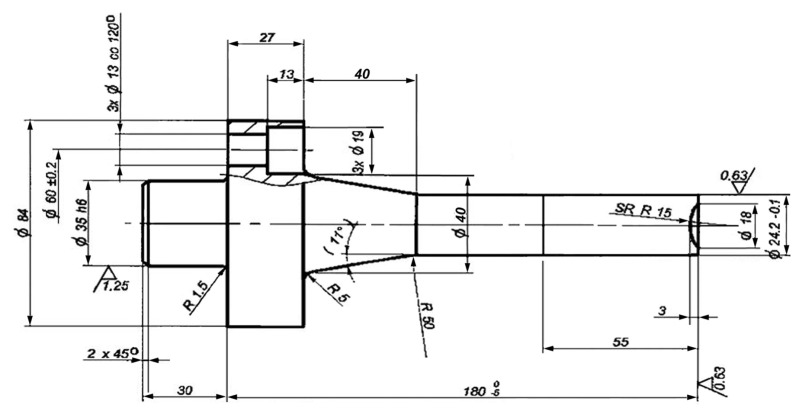
Punch made of 37CrMoV5-1 steel (WCL) [23].

Another important factor affecting tool longevity is the condition and quality of the forging equipment itself, which directly influences its operational durability [38]. The implementation of the methods described in this study, both in evaluating and enhancing tool life, is expected to enable a more accurate analysis of the problem of premature tool failure and contribute to a substantial extension of the durability of the forging tool. As part of the study, the authors carried out a technological process that involved the burnishing of the punches (working surfaces) under actual production conditions [24,38], as illustrated in Figure 5 and Figure 6. Operational tests confirmed that the service life of burnished punches was approximately 30–40% longer compared to that of conventional unprocessed tools.

### 2.5. Forging Process Technology and Exploitation Conditions

In plastic forming processes, high friction resistance occurs. This causes an intensive wear of the toolwork surfaces and significantly affects the condition of the surface layer for certain products (stereometric properties: surface roughness, waviness, and load bearing capacity; mechanical properties: state of residual stresses, degree of reinforcement, and texture). The correct development of the technological process of die forging consists of designing and making appropriate so-called auxiliary and die cuts based on the following analysis:

– Shape of the forging (axially symmetrical, compact, elongated, and complex) and mass of the forging;
– Kinematics of material flow on various forging machines (hammers, presses, forging machines, and aggregate sets);
– Plastic and mechanical properties of the deformed material, taking into account the forging temperature (cold or hot);
– Matrix construction (open and closed).

**Figure 7:**
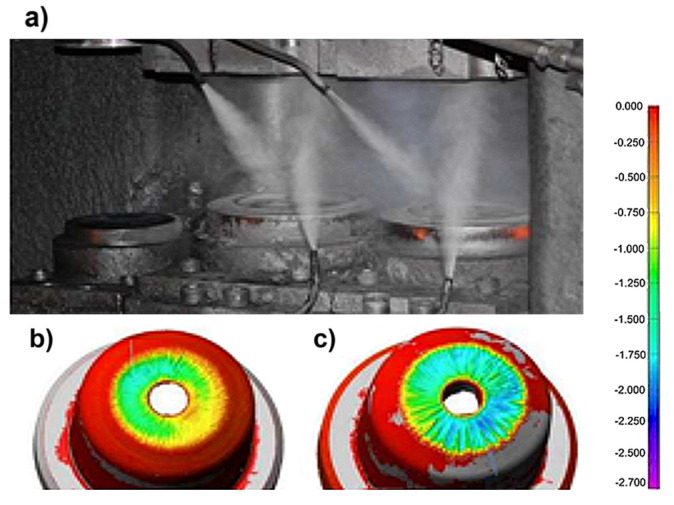
The appropriate lubrication method used on crank presses by manually setting the lubrication nozzles: (**a**) manual manner of lubricant supply, (**b**) tool wear in the case of non-uniform lubrication, and (**c**) tool wear in the case of uniform lubrication. Color maps (**b**,**c**) present the 3D surface deviation due to tool wear, as measured by optical scanning. The scale bar on the right represents the surface wear depth in millimeters, where red indicates minimal wear (0 mm), and dark blue indicates maximum material loss (up to ~−2.7 mm). Color maps (**b**,**c**) present the 3D surface deviation [mm] due to tool wear, as measured by optical scanning [14].

The correct development of the technology process for die forging also depends on the proper design of the forging [3]. Based on the conducted correct technological analysis, including the dominant degradation mechanisms (thermomechanical fatigue and abrasive wear) and detailed data on the process parameters (charge temperature), requirements that should be met by the material for forging, the durability of the dies can be improved. Another factor dependent on the operator (blacksmith) operating the machine directly and influencing premature wear of the equipment is failure to comply with the conditions and recommendations of the technological process. The necessary condition for proper preheating of the forging die is based on the recommendations of the technology developed for a given process. However, failure to comply with technology recommendations or their deliberate violation can often be encountered. According to the recommendations (of the relevant standards) and own research, the forging dies should be heated to a temperature of approximately 250 °C before starting work [38]. The method of heating dies using preheated forms is commonly applied in forging operations. However, in our professional experience, this method does not ensure adequate die heating or achieve the required process temperature. Insufficient die temperature results in reduced resistance to both static and dynamic loads. Furthermore, thermal shock caused by a significant temperature gradient between the heated workpiece and the cooler tool can lead to brittle fracture. The main objective of using various lubrication techniques and procedures is to reduce friction resistance, thereby reducing energy losses and the intensity of wear of the friction pair elements, allowing an increase in the durability and reliability of technical devices [64]. The complex mechanical and thermal loads that occur in the die forging processes and that are transferred by the forging tools (dies that work on presses, hammers, and forging machines) and the low durability of this group of tools impose particularly high requirements on the technological lubricants intended for use in these conditions [38]. A necessary condition to obtain favorable effects when using cooling and lubricating agents in pressing processes is the systematic supply of lubricant to the working space (cutout), and the lubrication cycle of the dies should be consistent with the forging cycle [47]. Figure 7 shows the appropriate lubrication method used on crank presses by manually setting the lubrication nozzles, according to the discretion and experience of the operator.

## 3. Principles of Manufacturing Dies from 42CrMo4 Steel by Surfacing Working Surfaces and Properties of Surfaced Layers on Forging Die Blanks

Another method of improving the durability of forging dies is to harden and face the working surfaces of forging tools, which can be done in various ways. Generally, a material with better properties is introduced into the surface layer to increase its resistance to wear [16]. The method mentioned above serves to weld regenerated dies or new tools. Many publications confirm the effectiveness of applied technologies [65]. Cobalt-based superalloy methods have been known for a long time and are popular [68]. Laser coating processes are proven, and we can find many publications [69]. The friction stir welding method is also often used [55]. Many publications present effective cladding methods that increase the durability of forging tools [5,10]. The authors presented a different method to improve the durability and performance of dies and hardfacing [38]. Economic considerations, especially the laboriousness of tool production and the large amounts of tool steel used, dictated the need to solve the problem of selecting materials in combination with the technology of making dies for forging on presses. It includes activities that improve the durability of press forging tools and, in particular, introduce dies made of alloy structural steel 42CrMo4 with hard-facing cutting surfaces instead of traditional tool steels for hot work. This requires the use of a hard-facing material with a properly selected chemical composition. The following types of wires have such properties: UTOP-38, F-812, and F-818, which have different mechanical and operational properties. The tests were carried out under industrial conditions at the Forge Plant “Glinik” (Poland). They covered pressing dies implemented in production. Forging processes were carried out on hydraulic presses LZK 1000, LZK 1600, and LKM 2500.

### Comparative Durability of Forging Dies Made of 37CrMoV5-1 (WCL) and 42CrMo4 Steel with Hard-Facing Surfaces of Cut-Outs

Example test results (authors’ concept) of three sets of dies used until their complete wear were traditionally made of 37CrMoV5-1 (WCL) steel and 42CrMo4 steel with hard-facing working surfaces. Zone hard-facing was used, with two types of wires: F-812 (on parts of the cuts exposed primarily to thermal fatigue (on parts of the cuts exposed mainly to thermomechanical fatigue)) and F-818 (in places particularly exposed to abrasive wear).

**Figure 8:**
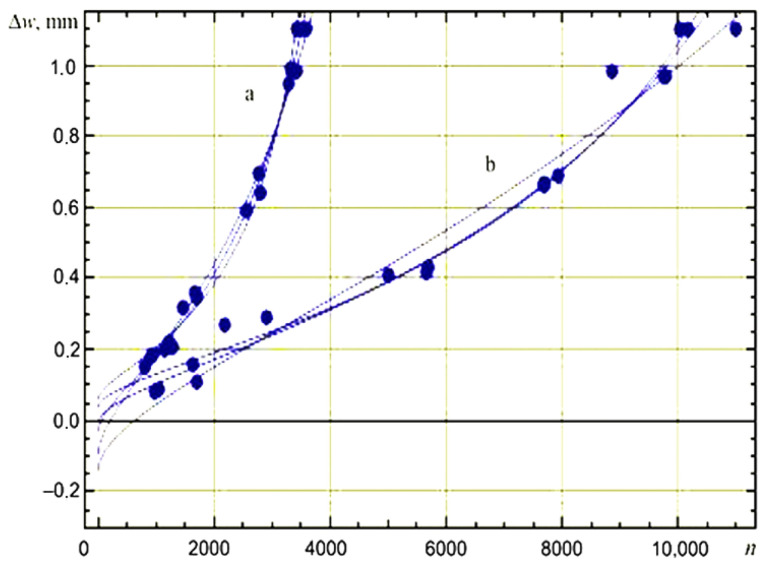
Comparative analysis of forging die wear curves indicating the durability of materials used for punches and dies: (a) made entirely of steel 42CrMo4; (b) composed of 42CrMo4 base material with welded working surfaces made of X37CrMoV5-1 steel. Δw—deviation in forging height; *n*—number of completed forgings [23].

The durability of the dies (Figure 8) made of 42CrMo4 steel with hard-working surfaces was (a) 10,160, 10,010, and 11,000, while for 37CrMoV5-1 steel (b) 3500, 3405, and 3589. All proposed and tested materials (UTOP38, F-812, and F-818) were used to reappear on cut surfaces. In selecting the material for surfacing in specific cases, the main considerations were the design and material of the forging and the type of cut (preliminary and finishing), trying to predict the dominant wear mechanisms of individual cuts or their fragments. The wear tests covered a very diverse range of forgings.

**Figure 9:**
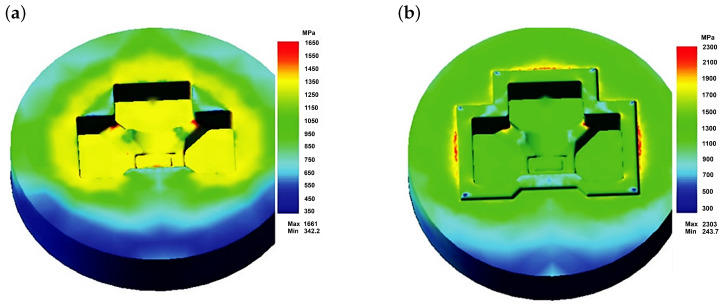
Numerically determined equivalent stress distributions according to the Huber–Mises hypothesis on the surfaces of the die for forging a tee [23]. (**a**) Lower half of the die; (**b**) upper half of the die.

**Figure 10:**
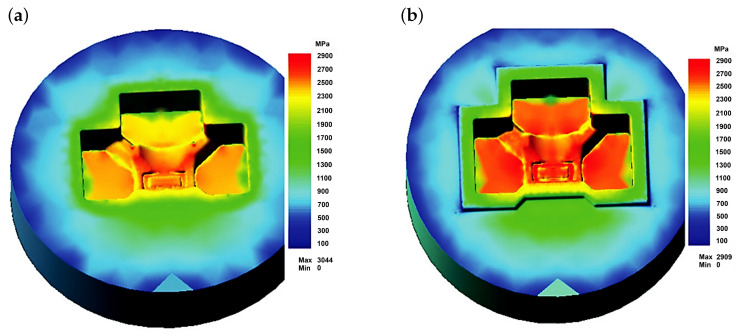
Numerically determined normal stress distributions on the surfaces of the die for forging a tee [23]: (**a**) lower half of the die; (**b**) upper half of the die.

**Figure 11:**
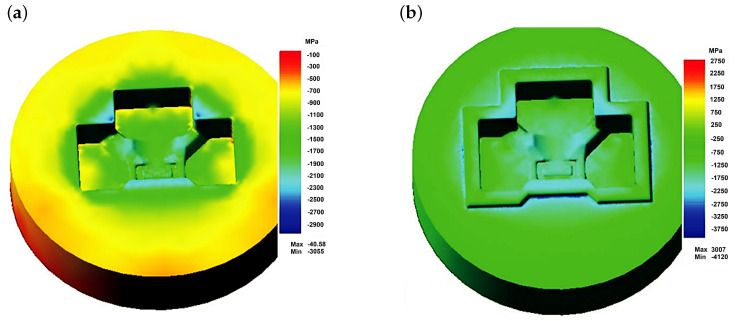
Numerically determined distributions of mean stress on the surfaces of the die for forging a tee [23]: (**a**) lower half of the die; (**b**) upper half of the die.

The authors described in the literature [38] the evaluation of the operating conditions of a specific die with a finishing impression to shape a tee forging. The finite element method (FEM) was used, combined with the solution of the Fourier equation for unsteady heat flows. The modeling of the forging process, combined with the determination of the parameters characterizing the loads and the die temperature, was carried out using the commercial program QForm 5.0, which is often used to analyze hot-plastic working processes and has been proven in similar applications. Examples of results from the FEM analysis for stresses are presented in Figure 9, Figure 10 and Figure 11.

Analyzing the results presented in Figure 9 and Figure 10, it can be stated that the loads on the die forming the finished forging are high. As expected, the highest values occur near the die corners. The level of equivalent stresses (Figure 9) should not cause significant plastic deformations in the die, provided that the die temperature does not exceed approximately 250 to 300 °C, while the unit pressures are high enough to expect rapid abrasive wear, especially of the upper half of the die (near the edges). This stress state is beneficial for the fatigue resistance of the material. Compressive (negative) stresses ”clamp” the material, closing potential cracks and impeding their propagation. As a result, fatigue damage accumulates more slowly, and tool life under cyclic load is extended. In contrast, tensile (positive) stresses tend to “pull apart” the material, promoting crack initiation and growth. Therefore, the predominant compressive stresses, indicated by the negative mean stress values, demonstrate that the die operates largely in a compressive elastic regime, which enhances its fatigue strength.

## 4. Discussion

This article provides a systematic review of various methods for regenerating and enhancing the durability of hot forging dies, covering material innovations, surface engineering, heat treatment, and process optimization. The findings are based both on the literature and the authors’ long-term industrial experience in a production environment.

This article provides a systematic review of various methods for regenerating and enhancing the durability of hot forging dies, covering material innovations, surface engineering, heat treatment, and process optimization. Currently, a wide range of die-refurbishment techniques is used, including various welding, cladding, and surface treatment processes. Each method presents specific advantages and limitations depending on the type of damage, the die material, and the operating conditions [70,71,72].

The success of forging die regeneration is largely determined by the compatibility between the overlay and base materials. A promising approach involves replacing traditional hot-work tool steels, such as X37CrMoV5-1, with alloy structural steels such as 42CrMo4 in combination with surfacing. Industrial trials in Forge Plant “Glinik” (Poland) have shown that this strategy can increase the durability of the tool by 200–300%. This improvement is largely attributed to zone surfacing techniques, where specific filler materials—F-812 for thermomechanical fatigue and F-818 for abrasive wear—are selectively applied to match localized stress patterns. The application of these materials represents a significant technological innovation developed through the authors’ long-term experience in industrial conditions. All conclusions and practical recommendations presented in this review are based on extensive experimental work and implementation trials conducted by the authors over many years in a production environment.

Post-weld heat treatment (PWHT) at 250–300 °C enhances microstructural stability by minimizing retained austenite and controlling carbide precipitation. Nonetheless, material incompatibility remains a challenge; for instance, cobalt-based superalloys like Stellite 21, though highly wear-resistant, may crack in H13 steel dies due to coefficient of thermal expansion mismatch. FEM simulations (QForm 5.0) confirm that gradient transition layers can mitigate interfacial stresses.

Advanced surfacing technologies such as laser cladding produce extremely dense coatings with minimal heat-affected zones. For example, laser cladding has demonstrated 150% durability improvements when applied to Unimax steel dies. Hybrid methods, including plasma transferred arc (PTA) welding combined with cryogenic treatment at −196 °C, have reduced abrasive wear by 40% in 55NiCrMoV6 dies.

Surface engineering continues to be a critical factor in tool longevity. Burnishing techniques introduce compressive stresses (up to −800 MPa) and reduce surface roughness to Ra 0.1μm, extending tool life by 30–40%. Similarly, modern lubrication systems using automated nozzles offer superior graphite dispersion compared to manual application, decreasing wear by approximately 22%.

Process optimization strategies such as Six Sigma DMAIC have led to a 35% reduction in process variability, further minimizing die wear. Economic analyses show that regeneration methods are up to 60% more cost-effective than producing new dies, with return on investment typically achieved after only 2–3 production batches.

Despite these advances, certain limitations persist. AlN ceramic inserts show promise in reducing thermal fatigue, but their brittleness currently limits application in high-impact environments. Furthermore, existing FEM models often underestimate thermal softening at temperatures above 600 °C, pointing to the need for improved simulation fidelity.

This review synthesizes these advances and challenges, emphasizing the interplay of materials, technology, and process control in the regeneration of forging dies. The findings presented are not only grounded in a comprehensive literature overview but are also directly drawn from the authors’ extensive industrial practice and research experience.

## 5. Conclusions

Hot forging die refurbishment has emerged as a multi-faceted domain involving material substitution, precision surfacing, and process engineering. Based on the authors’ long-term industrial research and implementation experience, replacing conventional hot-work tool steels with alloy structural steels such as 42CrMo4 in combination with engineered welded overlays has proven to significantly extend tool life under real production conditions. These findings stem from extensive trials and evaluations performed in a heavy forging plant environment. Ensuring compatibility between deposited and base materials remains essential to prevent failure modes such as cracking or delamination.

Our field experience confirms that zone-specific surfacing—tailoring filler materials to dominant wear mechanisms (e.g., F-812 for thermomechanical fatigue and F-818 for abrasive wear)—can significantly improve localized durability. These filler materials were selected and verified over years of systematic observation and wear analysis in forging operations. Similarly, laser cladding and hybrid techniques, including cryogenic post-treatment, have demonstrated excellent performance in minimizing distortion and extending the service life of refurbished dies.

Post-treatment methods such as burnishing, in conjunction with advanced coatings and sub-zero tempering, further enhance surface properties and improve structural resilience. Moreover, process optimization—via controlled forging parameters, tool geometry design, and statistical quality tools—has led to measurable improvements in die reliability and repeatability, as validated through in-process monitoring and operational data collection.

An integrated regeneration strategy, combining material compatibility, advanced surfacing methods, and optimized heat treatment, is essential for durable and cost-effective tool performance. Our research and industrial implementation show that such approaches not only prolong tool lifespan but also support sustainable manufacturing by reducing raw material consumption, energy usage, and overall production costs.

Looking forward, we believe that future work should emphasize the development of novel materials—such as ceramic-reinforced overlays—for extreme thermal and mechanical conditions and advance hybrid technologies that combine precision surfacing with AI-based predictive wear models. Additionally, efforts toward the cross-sector standardization of die regeneration protocols will be vital for broader industrial adoption.

In summary, this review combines a synthesis of current knowledge with practical insights derived from the long-standing operational experience in forging die maintenance and regeneration. The synergy of material innovations, surface engineering, and process control forms a robust framework for improving tool longevity and advancing sustainable manufacturing practices.

## Data Availability

No new data were created or analyzed in this study. Data sharing is not applicable to this article.

## Abbreviations

In this manuscript, the following abbreviations for various steel grades, designations, and proprietary materials are used:

**Abbreviation**	**Description**
X37CrMoV5-1	Hot-work tool steel according to EN ISO 4957
1.2343	DIN designation equivalent to X37CrMoV5-1
H11	AISI designation equivalent to X37CrMoV5-1
42CrMo4	Alloy structural steel used as a die material substitute
1.7225	DIN designation for 42CrMo4
55H3SMF	Polish Cr-Mo-V steel with sulfur for improved machinability
4340 mod.	AISI equivalent to 55H3SMF (with V, S)
41HMFS	Polish equivalent to 42CrMo4 with added V and S
4140 mod.	AISI equivalent to 41HMFS (with V, S)
50H2SF	Polish Cr-V spring or structural steel with sulfur
50CrV4	EN designation for spring steel similar to 50H2SF
1.8159	DIN designation for Cr-V spring steel
6150	AISI equivalent to 50H2SF
F-818	Fully martensitic wire, ideal for high-wear and abrasive conditions
F-812	Martensitic-bainitic wire with surface ferrite, suitable for thermo-mechanical fatigue
UTOP38	Dual-phase wire with rich ferritic content, effective under extreme fatigue
